# Dye decolorization and detoxification potential of Ca-alginate beads immobilized manganese peroxidase

**DOI:** 10.1186/s12896-015-0227-8

**Published:** 2015-12-10

**Authors:** Muhammad Bilal, Muhammad Asgher

**Affiliations:** Industrial Biotechnology Laboratory, Department of Biochemistry, University of Agriculture, Faisalabad, Pakistan

**Keywords:** *Ganoderma lucidum*, Manganese peroxidase, Immobilization, Reactive dyes, Decolorization, Cytotoxicity

## Abstract

**Background:**

In view of compliance with increasingly stringent environmental legislation, an eco-friendly treatment technology of industrial dyes and effluents is a major environmental challenge in the color industry. In present study, a promising and eco‐friendly entrapment approach was adopted to immobilize purified manganese peroxidase (MnP) produced from an indigenous strain of *Ganoderma lucidum* IBL-05 on Ca-alginate beads. The immobilized MnP was subsequently used for enhanced decolorization and detoxification of textile reactive dyes).

**Results:**

MnP isolated from solid-state culture of *G. lucidum* IBL-05, presented highest immobilization yield (83.9 %) using alginate beads prepared at optimized conditions of 4 % (w/v) sodium alginate, 2 % (w/v) Calcium chloride (CaCl_2_) and 0.5 mg/ml enzyme concentration. Immobilization of MnP enhanced optimum temperature but caused acidic shift in optimum pH of the enzyme. The immobilized MnP showed optimum activity at pH 4.0 and 60 °C as compared to pH 5.0 and 35 °C for free enzyme. The kinetic parameters *K*_*m*_ and *V*_*max*_ of MnP were significantly improved by immobilization. The enhanced catalytic potential of immobilized MnP led to 87.5 %, 82.1 %, 89.4 %, 95.7 % and 83 % decolorization of Sandal-fix Red C_4_BLN, Sandal-fix Turq Blue GWF, Sandal-fix Foron Blue E_2_BLN, Sandal-fix Black CKF and Sandal-fix Golden Yellow CRL dyes, respectively. The insolubilized MnP was reusable for 7 repeated cycles in dye color removal. Furthermore, immobilized MnP also caused a significant reduction in biochemical oxygen demand (BOD) (94.61-95.47 %), chemical oxygen demand (COD) (91.18-94.85 %), and total organic carbon (TOC) (89.58-95 %) of aqueous dye solutions.

**Conclusions:**

*G. lucidum* MnP was immobilized in Ca-alginate beads by entrapment method to improve its practical effectiveness. Ca-alginate bound MnP was catalytically more vigorous, thermo-stable, reusable and worked over wider ranges of pH and temperature as compared to its free counterpart. Results of cytotoxicity like hemolytic and brine shrimp lethality tests suggested that Ca-alginate immobilized MnP may effectively be used for detoxification of dyes and industrial effluents.

## Background

Synthetic dyes are extensively used in textile, paper, cosmetic, pharmaceutical, dyeing and printing industries. The disposal of dye containing industrial effluents into receiving water bodies’ triggers serious environmental and health hazards [1–5]. Among various groups of dyes, reactive dyes are the most problematic, as their complex aromatic structures are resistant to bio-degradation [6]. Such scenario has created great concern among industrialists and scientific community for their economic treatment and safe disposal. Various physico-chemical processes, such as chemical coagulation/flocculation, membrane separation, ultrafiltration, ion exchange, froth flotation, reverse osmosis and adsorption have been described for decolorization of reactive dyes. However, due to many drawbacks like high cost, low efficiency, secondary pollution, residues waste problem and inapplicability to treat a wide variety of dyes, these methods are regarded economically un-acceptable for large-scale effluent treatment [5, 7]. These facts certainly demand the development of an efficient, cost effective and green technology for decolorization and detoxification of dyes and industrial effluents. Biological approach using ligninolytic system of white rot fungi (WRF) seems to be the most potential alternative than traditional physico-chemical methods [8–10].

*Ganoderma lucidum* is an efficient lignin degrading WRF that produces considerable activities of ligninolytic enzymes, particularly manganese peroxidase (MnP). Among the potential applications of MnP are bioremediation, biomass delignification, biopulping, biosensor development, textile finishing and wine stabilization [2, 11]. Despite their great potential, the use of native microbial enzymes suffers certain restrictions under industrial process conditions. To circumvent these limitations, several strategies including mutations, genetic engineering, chemical modifications of amino acid residues and immobilization have been suggested. Enzyme immobilization appears to be an attractive approach to develop efficient biocatalyst with improved performances such as enhanced resistance to thermal and chemical inactivation, remarkable storage and operational stabilities, short response time and high reproducibility [12, 13].

In recent past, various immobilization supports like macroporous exchange resins, Ca-alginate beads, chitosan beads, polyvinyl alcohol, nanoporous silica gel, polyacrylamide and hydrophobic sol-gels have been used for MnP immobilization [14]. Entrapment in calcium alginate beads is of particular interest because of very mild and simple preparation conditions, non-toxicity, low cost and best performance [15]. Indeed, Alginate is a natural anionic poly-saccharide composed of repeated units of α-L-guluronic acid and *β*-D-mannuronic acid residues. Alginate supports are usually prepared by cross linking of guluronic acid with mannuronic acid residues in the presence of divalent cations like Ca^2+^, Ba^2+^, Co^2+^ [16].

In the present study, MnP from *G. lucidum* IBL-05 was immobilized into Ca-alginate beads and the conditions for immobilization and characterization of free and immobilized enzyme were investigated. The reusability, thermal and storage stability of immobilized MnP were also studied and compared with free enzyme. In addition, the capability of both free and immobilized MnP to decolorize different textile dyes (Sandal reactive dyes) was assessed. The treatment efficiency was evaluated on the basis of decolorization, water quality parameters (BOD, COD, TOC) and cytotoxicity (erythrocytes lysis and brine shrimp lethality) reduction.

## Methods

### Chemicals and dyes

Coomassie Brilliant Blue G-250, sodium dodecylsulphate, Sephadex G-100, N, N, N΄, N΄-tetra-methylethylenediamine, *ß*-mercaptoethanol, trizma base, sodium alginate, calcium chloride dihydrate (CaCl_2_. 2H_2_O) and glutaraldehyde from Sigma chemicals (St. Louis, USA) were supplied by local suppliers. Triton X-100 and cyclophosphamide were purchased from Merck (Germany) and Scharlau (Spain), respectively. All chemicals and reagents were of analytical grade and used without further purification. Five Sandal Reactive textile dyes including Sandal-fix Red C_4_BLN, Sandal-fix Turq Blue GWF, Sandal-fix Foron Blue E_2_BLN, Sandal-fix Black CKF and Sandal-fix Golden Yellow CRL were generously gifted by Sandal Dye-stuffs Faisalabad, Pakistan. Characteristics of selected dyes have been presented in Table 1.

**Table 1:** Characteristics of Sandal reactive dyes used in immobilized MnP catalyzed decolorization studies

Dyes	Color	$\lambda$_ max_	Class	CI Number
Sandal-fix Red C_4_BL	Red	540	Reactive	Reactive Red 195A
Sandal-fix Turq Blue GWF	Blue	664	Reactive	Reactive Blue 21
Sandal-fix Golden Yellow CRL	Yellow	414	Reactive	Reactive Yellow 145A
Sandal-fix Black CKF	Black	598	Reactive	Mixture
Sandal-fix Foron Blue E_2_BLN	Foron Blue	560	Reactive	Not known

CI: Color Index

### Preparation of lignocellulosic substrate

Agro-industrial waste material wheat bran collected from a local wheat mill of Faisalabad, Pakistan was sun dried followed by oven drying at 60 °C to constant weight. The dried substrate was pulverized to 0.45-0.90 mm meshes in grinder (Ashraf Herbal Laboratories**,** Faisalabad) and stored in airtight plastic jars.

### Microorganism and inoculum development

A pure culture of locally isolated fungal strain *G. lucidum* IBL-05 was maintained on potato dextrose agar (PDA) slants and preserved in the culture collection of Industrial Biotechnology Laboratory, Department of Biochemistry, University of Agriculture; Faisalabad). Kirk′s basal medium supplemented with 1 % (w/v) Millipore filtered sterile glucose solution was used as inoculum medium [11]. The medium constituents were (g/L): ammonium tartrate, 0.22; KH_2_PO_4,_ 0.21; MgSO_4_ · 7H_2_O, 0.05; CaCl_2_ · H_2_O, 0.01; thiamine, 0.001 and tween‐80 (10 ml/L). The pH of the medium was adjusted to 4.5 using 1M HCL/1M NaOH and sterilized in autoclave (Sanyo, Japan) at 121 °C for 15 min. *G. lucidum* IBL-05 from slant culture was aseptically transferred to the sterilized medium in laminar air flow (Dalton, Japan). The inoculated flask was incubated at 30 °C for 5 days in an orbital shaker (Sanyo-Gallenkamp, UK) with continuous shaking (120 rpm) to obtain homogenous spore suspension (1 × 10^7^ spores/ml). The spore counting was performed using haemocytometer (Sigma-aldrich, USA).

### Production and extraction of MnP

Erlenmeyer flasks (triplicate), each containing 5 g wheat bran were moistened with Kirk’s basal nutrient medium (66 % w/w) at pH 4.5 and cotton plugged. The flasks were sterilized in laboratory scale autoclave (Sanyo, Japan) and inoculated with 5 ml (1 × 10^7^ spores/ml) freshly prepared inoculums of *G. lucidum* IBL-05. The inoculated flasks were allowed to ferment in still culture incubator (Sanyo, Japan) at 30 °C for 5 days [19]. After 5 days, 100 ml of distilled water was added to the fermented biomass and shaken for 30 min at 120 rpm (Sanyo-Gallenkamp, UK). The contents of flasks were filtered, centrifuged (Eppendorf 5415C, Germany) and clear supernatants thus obtained were analyzed for MnP activity.

### Determination of MnP activity and protein contents

MnP was assayed by a previously reported assay method [17]. Assay mixture (2.6 ml) containing 1ml of 1mM MnSO_4_, 1 ml of 0.05 M sodium malonate buffer (pH 4.5), 0.5 ml of H_2_O_2_ and 0.1 ml of enzyme solution was incubated at 25 °C for 10 min. Absorbance of each sample was measured at 270 nm (ε_270_ = 11570 M cm^−1^) in double beam UV/Visible spectrophotometer (HALO DB 20). Activity assay for immobilized enzyme was performed in the same conditions used for free enzyme, except that the reaction were maintained with stirring, and interrupted by separation of enzyme-immobilized beads from the reaction mixture by filtration in a Buchner funnel before the spectrophotometric readings. One unit of MnP activity (U) is defined as the amount of enzyme capable of producing 1 *μ*mol of product in one min under the specified reaction conditions. The values obtained in the blank reactions were discounted from all readings. Bradford micro-assay [18] was followed for the determination of total protein contents in enzyme extract before and after every purification step. 10 μl of enzyme sample was added to 1 ml of Bradford reagent followed by incubation at 37 °C for 15 min. The absorbance was read at 595 nm and protein was estimated from standard curve using Bovine Serum Albumin (BSA) as standard.

### Purification of MnP

Four step purification procedure involving ammonium sulphate fractionation, dialysis, diethyl amino ethyl (DEAE) cellulose ion exchange and G-100 Sephadex gel filtration chromatography was employed for the purification of MnP. Crude MnP extract from *G. lucidum* IBL-05 was saturated by gradual addition of ammonium sulfate (up to 65 %), centrifuged and pellets were dissolved in 50 mM Sodium Malonate buffer (pH 4.5), and dialyzed overnight against the same buffer. The dialyzate was subjected to ion exchange chromatography using DEAE-cellulose column. The column was equilibrated with phosphate buffer (pH 6.5) for 24 h and eluted with 0 to 1.0 M linear gradient of NaCl in 50 mM malonate buffer at a flow rate of 0.5 ml/min. The MnP active fractions were pooled and loaded on Sephadex-G-100 column (10 × 300 mm). A 50 mM malonate buffer was used for elution (flow-rate 0.3 ml/min) and positive fractions were collected, pooled and stored at -20 °C [19].

### Immobilization of MnP

The purified MnP was mixed with 4 % sodium-alginate solution and 50 mM sodium malonate buffer (pH 4.5) in 1:1:1 ratio [20]. To this mixture, 0.1 ml gluteraldehyde solution (0.8 %; v/v) was added and mixed gently. The resultant solution was extruded drop-wise into CaCl_2_ (200 mM) solution using a syringe needle to prepare uniform size beads. The beads were transferred to fresh CaCl_2_ solution and incubated for 30 min at 4 °C. After 30 min of hardening, the beads were separated from CaCl_2_ solution by vacuum filtration and washed on a filter thrice with distilled water and finally with 50 mM sodium malonate buffer (pH 4.5). All the Ca-alginate beads were dried at -70 °C under vacuum (0.1 mm Hg) for 15 h followed by vacuum-drying at room temperature for 3 h. Immobilization (%) was determined according to the equation indicated below:

$$ \%\;\mathrm{immobilization}=\frac{\mathrm{Total}\;\mathrm{activity}\;\mathrm{of}\;\mathrm{immobilized}\;\mathrm{enzyme}}{\mathrm{Total}\;\mathrm{activity}\;\mathrm{of}\;\mathrm{free}\;\mathrm{enyme}}\times 100 $$

### Characterization of free and immobilized MnP

The free and Ca-alginate beads immobilized MnP were characterized by studying the following parameters:

### Effect of pH

In order to determine the pH optima for free and immobilized MnP, the reaction mixture was incubated for 15 min in buffers of pH 3.0-10.0. After incubation, the enzyme assay was performed using standard assay protocol. The buffers used were: tartrate-buffer, pH 3.0; sodium-malonate buffer, pH 4.0; citrate-phosphate, pH 5.0, pH 6.0; sodium-phosphate, pH 7.0, pH 8.0 and carbonate-buffer of pH 9.0 and 10.0.

### Effect of temperature

The free and immobilized MnP activity was tested at different temperatures (30, 35, 40, 45, 50, 55, 60, 65 and 70 °C) for 1h at optimum pH before running the routine MnP assay.

### Effect of substrate concentration: Determination of kinetic parameters

Effect of substrate concentration on activities of free and immobilized MnP was studied at optimum pH and temperature using varying concentrations of MnSO_4_ ranging from 0.1-1.0 mM. Lineweaver’s-Burk reciprocal plots were constructed between 1/S and 1/V_0_ and kinetic parameters of Michaelis-Menten (*K*_m_ and *V*_max_) were determined.

### Decolorization of sandal reactive dyes

A set of five Sandal reactive textile dyes was selected to investigate the decolorization potential of free and Ca-alginate beads immobilized MnP. Free and immobilized MnPs were transferred to 250-ml cotton plugged Erlenmeyer flasks (triplicate) containing 100 ml of individual dye solution (0.1 mg/ml), 1 mL of 1 mM MnSO4, 0.1 mM H_2_O_2_ and Na-malonate buffer (50 mM; pH 4.5). Flasks were incubated at 35 °C on rotary shaker at 120 rpm for 12 h. After 12 h, the contents of flasks were filtered, centrifuged (8,000× *g*, 10 min) and residual dye concentration was monitored at respective wavelengths of maximum absorbance (CE Cecil 7200, Germany) [3]. The decolorization efficiency of free and immobilized MnP for each dye was calculated using the relation given in equation 2. Where A*i* and A*t* are representing absorbance at zero and time *t*.

2 $$ \mathrm{Decolorization}\;\left(\%\right)=\frac{Ai-At}{Ai}\times 100 $$

## Stability studies

### Thermal and storage stability

Thermal stability was assayed by incubating free and immobilized MnPs simultaneously at 60 °C for 240 min. Storage stability of both free and Ca-alginate immobilized MnPs was investigated for a period of 60 days at 25 °C and the residual activity was monitored from time to time under standard assay conditions described above. Enzyme activity prior to incubation was defined as 100 %.

### Reusability

Seven decolorization cycles of 12 h each were performed to test the reusability of Ca-alginate immobilized MnP. At the end of each dye-decolorization cycle, Ca-alginate beads were filtered, washed three times with sodium malonate buffer (50 mM) and replaced with fresh aqueous dye solutions. The activity of freshly prepared beads in the first run was defined as 100 %.

### Water quality parameters analysis

The water quality parameters such as BOD, COD and TOC were measured for maximally decolorized dye solutions. BOD and COD values were monitored using BOD and COD meters (Lovibond, water testing systems). For TOC measurement, 2N K_2_Cr_2_O_7_ (1 ml) and H_2_SO_4_ (1.6 ml) were taken in digestion flask containing dye samples (4 ml) and the contents were digested for 1.5 h at 110 °C, cooled and absorbance was monitored at 590 nm.

### Cytotoxicity evaluation

To evaluate free and Ca-alginate immobilized MnPs effect on cytotoxicity reduction, erythrocytes lysis and brine shrimp lethality tests were used [5]. The cytotoxicity of maximally decolorized dye solutions was investigated.

### Statistical analysis

Mean and standard error (SE) values of the results from three replicates were calculated using Microsoft Excel-software (Microsoft) and the standard error (SE) values have been displayed as Y‐error bars in figures.

## Results and discussion

### MnP production and purification

A large magnitude of extracellular MnP (717.7 ± 2.3 U mL^−1^) was produced from a locally isolated WRF strain *G. lucidum* IBL-05, grown on wheat bran as described in “Materials and Methods”. The cell-free crude MnP extract was purified by using a four step procedure involving ammonium sulphate fractionation, dialysis, ion exchange and gel filtration chromatography as summarized in Table 2 [21]. Previously, the purification of various fungal protease, cellulases and ligninolytic enzymes from *Aspergillus niger*, *Trichoderma harzianum*, *Pleurotus ostreatus* IBL‐02, *Trametes versicolor* IBL‐04 and *Schizophyllum commune* IBL‐06 using the similar 4 step purification protocol has also been reported [1, 19, 22, 23].

**Table 2:** Purification of extracellular MnP from *G. lucidum* IBL-05

Procedure	Total volume (mL)	Total protein (mg)	Total activity (UI)^a^	Specific activity^b^ (UI mg^−1^)	Purification^c^ (fold)
Crude extract	300	1302.3	215220	165.3	1
Ammonium Sulphate ppt.	22	43.9	13501.4	307.5	1.86
Dialysis	21	39.2	12559.9	320.4	1.94
DEAE-cellulose	12	6.37	6811.2	1069.3	6.47
Sephadex G-100	9	2.9	4599.1	1585.9	9.6

^a^UI is defined as the amount of enzyme converting 1 μmol of substrate per minute.

^b^Specific activity = UI per mg protein.

^c^Purification fold= Specific activity at given step/Specific activity of initial extract.

### Immobilization of MnP

**Fig. 1:**
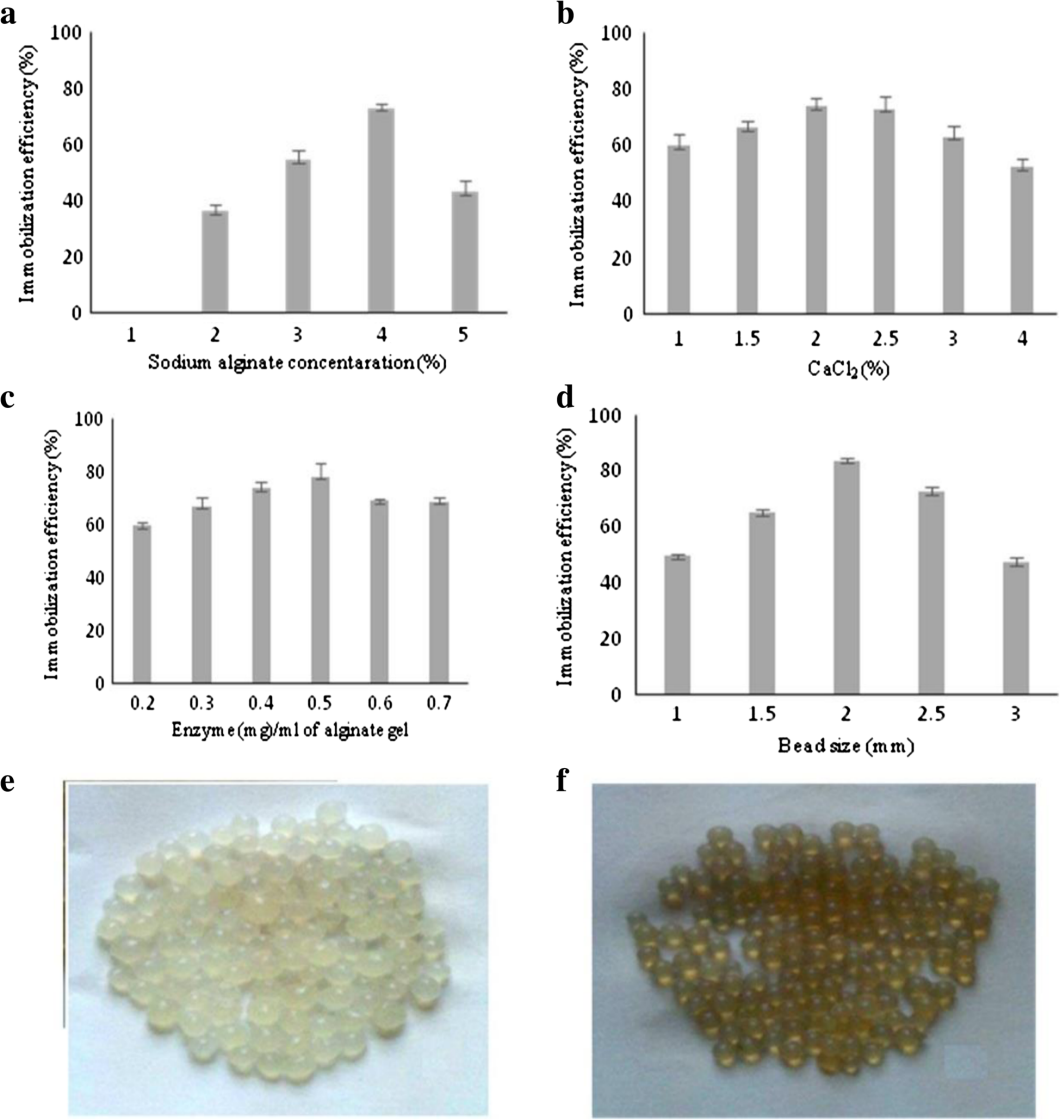
(**a**) Effects of alginate concentration. Immobilization conditions: MnP enzyme (0.2-0.7 mg/ml alginate gel), CaCl_2_ solution (1 % w/v); at 1 % sodium alginate concentration no beads were formed. (**b**) Effects of CaCl_2_ concentration. Immobilization conditions: MnP solution (0.2-0.7 mg/ml alginate gel), alginate solution (4 % w/v). (**c**) Effects of ratio of enzyme to alginate. Immobilization conditions: alginate (4 % w/v), CaCl_2_ (2 % w/v) (**d**) Effect of different alginate beads size. Immobilization conditions: alginate (4 % w/v), CaCl_2_ (2 % w/v), MnP solution (0.5 mg/ml alginate gel) (**e**) Control Ca-alginate beads (**f**) Ca-alginate beads with immobilized MnP

Different experimental conditions were optimized for development of stable Ca-alginate beads. Varying concentrations of sodium alginate (1-5 % (w/v)) were tried to get beads with desired mechanical strength. Sodium alginate at a concentration of 4 % (w/v) registered the highest immobilization efficiency of 73.15 % (Fig. 1a).

A Na-alginate concentration of 3 to 4 % has also been found suitable for protease immobilization [24]. At lower concentrations, the entrapped MnP leached out due to less tightly cross-linked alginate gel and greater pore size of the beads. Likewise, at increased Na-alginate concentration (> 4 %), the immobilization efficiency again declined due to decreased gel porosity, high viscosity of the beads and substrate diffusion restrictions [4, 25]. Similarly, higher the concentration of sodium alginate, the smaller the pore size of the beads leading to reduced immobilization efficiency [4].

The effect of CaCl_2_ concentration (1 to 2 % w/v) at 4 % fixed Na-alginate concentration on immobilization efficiency was also investigated (Fig. 1b). Because alginate anions and Ca^2+^ cations are cross-linked to form Ca-alginate beads, it is expected that an increase in alginate or CaCl_2_ concentration will render it difficult for *G. lucidum* MnP to escape out from the gel network. However, the effect of CaCl_2_ on immobilization yield was very small in the tested range of 1-4 %. The excessive Ca^2+^ ions might not affect the formation of gel-like networks [4].

In next step, different MnP concentrations (0.2-0.7 mg/ml) were used. The results presented in Fig. 1c indicated a direct dependency of immobilization efficiency with increasing enzyme concentration up to 0.5 mg MnP per ml of alginate solution. Beyond this concentration, saturation in capacity of immobilization support occurs and immobilization efficiency declined slightly. Higher protein concentrations did not yield better immobilization, while the lower concentrations were not enough to saturate most of the enzyme binding sites on activated matrix [4].

The alginate beads size may be the most important parameter for optimal MnP immobilization. It was predicted that enzyme entrapped in smaller size beads showed higher immobilization efficiency due to reduced substrate transfer resistance. Alginate beads of different sizes (1-3 mm) were generated by changing the size of needle through which a mixture of MnP and alginate was dropped into CaCl_2_ solution. Fig. 1d shows that 2 mm size beads furnished greater immobilization efficiency. As expected, the increased bead size leads to diminished immobilization yield of entrapped MnP. Other researchers have also reported the decline in activity of immobilized enzyme with increasing beads size due to mass transfer resistance [26].

The optimal immobilization conditions were 4 % (w/v) sodium alginate, 2 % (w/v) CaCl_2_ and 0.5 mg/ml enzyme concentration. Beads (2 mm diameter) developed under such conditions exhibited the maximum immobilized yield of 89.3 ± 2.4 %.

### Characterization of free and Ca-alginate immobilized MnP

A significant change in biochemical and kinetic parameters upon immobilization appraised the success of immobilization protocol. It is important to note that several factors can significantly affect the catalytic potential of ligninolytic enzymes. Among them, pH, temperature and substrate concentration are the most relevant factors that not only affect enzyme activity but also reduce the stability if these are not at their optimum [27].

### Optimum pH

**Fig. 2:**
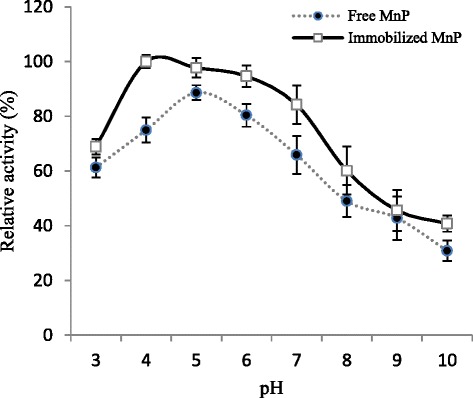
Effect of pH on activities of free and Ca-alginate immobilized MnP

The optimum pH for free and Ca-alginate immobilized MnP were found to be 5.0 and 4.0, respectively. Immobilization caused broadening of pH for immobilized MnP compared with free enzyme (Fig. 2). Moreover, Ca-alginate immobilized MnP displayed greater pH-stability towards the acidic and alkaline changes in medium than its free counterpart. In previous reports, the maximum activities of MnPs from different WRF were observed to shift from alkaline to acidic pH [28]. Asgher and co-workers, [29] also observed similar shift in optimum pH for immobilized MnP.

### Optimum temperature

**Fig. 3:**
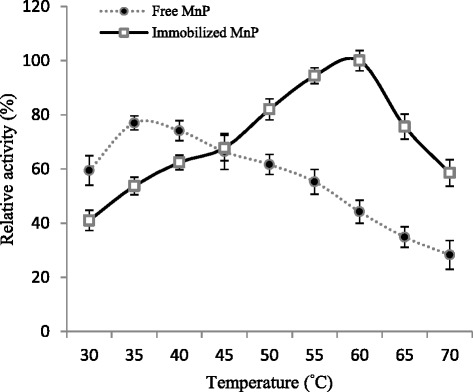
Effect of temperature on activities of free and Ca-alginate immobilized MnP

The temperature-activity profiles of both native and Ca-alginate bound MnP have been presented in Fig. 3. The optimum temperature for soluble MnP was 35 °C but immobilized MnP exhibited highest activity at 60 °C. The hydrophobic and other secondary interactions might impair conformational flexibility requiring higher temperature for the enzyme molecule to reorganize and attain a proper conformation in order to maintain its reactivity [30, 31]. Similar displacement in optimum temperature for immobilized enzyme has been reported in earlier studies where free MnP from *Irpex lacteus* showed maximum activity at 40 °C [32] and Ca-alginate bound ligninolytic enzyme was optimally active at 80 °C [7].

### Effect of substrate concentration: Determination of *K*m and V*max*

**Fig. 4:**
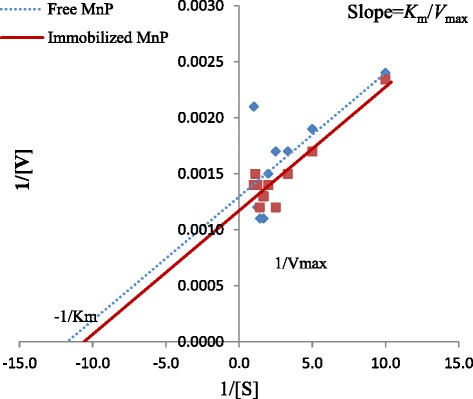
Determination of *K*_m_ and *V*_max_ by Lineweaver Burk Plot: Standard quartz (Si O_2_) cuvettes of 1 mm path length were used to calculate the values of kinetic parameters. Both free and immobilized MnP were incubated for 15 min at 30 °C in sodium malonate buffer of pH 4.5 before carrying out standard enzyme assay protocol. Lineweaver‐Burk (Double reciprocal) plots were generated with Microsoft Excel Windows updated version 7 via nonlinear regression analysis using different concentrations (0.1-1.0 mM) of manganese sulphate as substrate at optimum pH and temperature conditions

Ca-alginate immobilized MnP showed lower substrate affinity, as confirmed by its higher *K*_m_ value (*K*_m_; 82 mM) than that of free enzyme (*K*_m,_ 65.64 mM) (Fig. 4); this was in agreement with other investigators reporting decreased affinity of immobilized MnP for substrate [29]. On the other hand, catalytic efficiency of immobilized MnP was enhanced (*V*_*max*;_ 743 U/ml) as compared to free MnP (*V*_max_; 640 U/ml) that signifies the feasibility of immobilized system for myriad industrial applications. Similar findings indicating an increased *K*_m_ and *V*_max_ values following entrapment in Ca-alginate beads have also previously been reported [33].

### Dyes decolorization with free and immobilized MnP

**Fig. 5:**
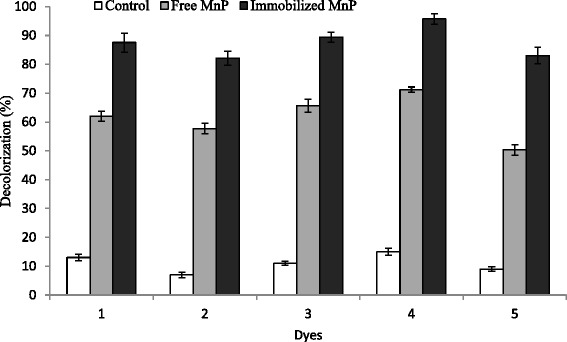
Decolorization of Sandal reactive dyes (*1*) Sandal-fix Red C_4_BLN (*2*) Sandal-fix Turq Blue GWF (*3*) Sandal-fix Foron Blue E_2_BLN (*4*) Sandal-fix Black CKF (*5*) Sandal-fix Golden Yellow CRL by free and Ca-alginate immobilized MnP. MnP decolorization reaction system contained; Na-malonate buffer (50 mM; pH 4.5), 0.1 mg/ml each dye solution, 10 mL free and 5 g of Ca-alginate-entrapped bio-catalyst (MnP),1 mL of 1 mM MnSO_4_ as MnP mediator, 0.1 mM H_2_O_2_ to a total volume of 100 mL. The flasks were incubated at 120 rpm for 12 h. Untreated dye solution containing all reagents and Ca-alginate beads without entrapped MnP enzyme was used as control

The dye-decolorizing potential of free and immobilized MnP from *G. lucidum* was investigated against a set of five Sandal reactive dyes and results, thus obtained are presented in Fig. 5 (decolorization, %). The color removal pattern with free and immobilized MnP was found to be significantly different. All tested dyes were more efficiently decolorized by immobilized MnP as compared to its free counterpart. In the presence of MnSO_4_ (1 mM) as redox mediator, a maximum decolorization efficiency of 61.9 %, 57.6 %, 65.5 %, 71.2 % and 50.3 % for Sandal-fix Red C_4_BLN, Sandal-fix Turq Blue GWF, Sandal-fix Foron Blue E_2_BLN, Sandal-fix Black CKF and Sandal-fix Golden Yellow CRL, respectively was achieved after 12 h with free MnP, whereas it increased to 87.5 %, 82.1 %, 89.4 %, 95.7 % and 83 %, respectively for Ca-alginate immobilized MnP. Immobilization prevents enzyme washouts and allows a high enzyme concentration to be maintained in a continuous reactor. Since the catalytic stability is often improved by immobilization, enzymes may degrade a higher concentration of toxic compounds then their free counterpart. Slight agitation (120 rpm) of the beads favored rapid degradation, presumably caused by increased oxygen supply to the beads and ease translocation of substrate and products. Similar findings regarding Ca-alginate immobilized ligninolytic enzymes and textile dyes decolorization were reported by Vishwakarma et al. [34] who achieved 99 % decolorization efficiency of immobilized MnP for azo dyes after 18 h incubation. Peralta-Zamora et al. [35] decolorized four synthetic dyes in the range of 5-55 % within 30 min and Kunamneni et al. [36] found 61-82 % decolorization of synthetic dyes in short contact time of 6 h.

Generally, it is expected that dye removal using Ca-alginate immobilized enzyme may be due to either enzymatic biodegradation or bioaccumulation/biosorption of the dye onto alginate beads [4]. In this study, to detect any possible removal of color due to dye adsorption onto the alginate beads, a control reaction with Ca-alginate beads without bound MnP was prepared. It was observed that the alginate beads became colored especially after contacting with dyes. However Ca-alginate beads were able to remove only 7-15 % color for all dyes. Thus, observation established that the predominant mechanism involved in dyes color removal was MnP biodegradation.

### Stability studies of immobilized MnP

#### Thermal stability

**Fig. 6:**
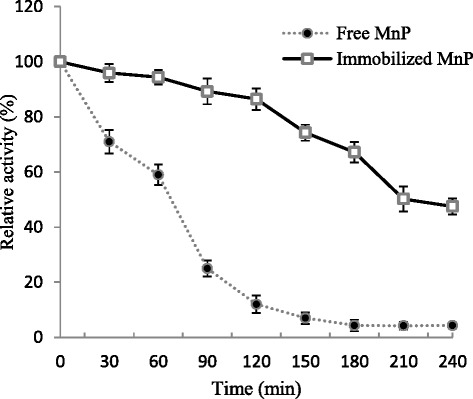
Thermo-stability of free and immobilized MnP. MnP immobilized Ca-alginate beads were incubated in Na-malonate buffer solution (50 mM; pH 4.5) at 60 °C for 240 min. The residual activities were determined at different time intervals. The experiments were conducted in triplicate

The thermal-stability of free and immobilized *G. lucidum* MnP was examined at 60 °C for different incubation times. After 120 min, the free and immobilized MnP retained 12.0 ± 3.8 % and 86.45 ± 3.2 % of their initial activities, respectively. The residual activities at 240 min were recorded to be 4.3 % ± 1.2 % for free MnP while 47.5 ± 2.9 % for immobilized MnP (Fig. 6). Similar to our findings, Daassi et al. [4] found that Ca-alginate immobilized *C. gallica* laccase showed 67 % residual activity after 210 min incubation at 55 °C. From the results, it can be inferred that immobilized MnP was more stable as compared to its free counterpart.

#### Storage stability

**Fig. 7:**
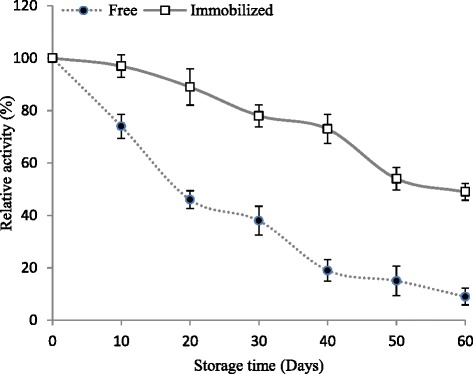
Storage stabilities of free and Ca-alginate immobilized MnP. Free and immobilized MnPs were kept at 25 °C for 60 days, and the activities were monitored from time to time under standard assay conditions. Enzyme activity prior to incubation was defined as 100 %. The experiments were performed in triplicate

The effect of storage on activities of free and entrapped MnP was investigated by incubating at room temperature (25 °C) for up to 60 days. Fig. 7 shows that, after 30 days of storage time, the free and immobilized MnP retained about 38.4 % and 77.2 % of their initial activities, respectively. Immobilization significantly enhanced the storage stability of Ca-alginate immobilized MnP that could be a valuable feature of this enzyme to be exploited for longer storage periods in industrial sectors. Previously, the activity of Ca-alginate immobilized laccase was found to be 60 % more than free laccase after 30 days storage [4].

#### Reusability

**Fig. 8:**
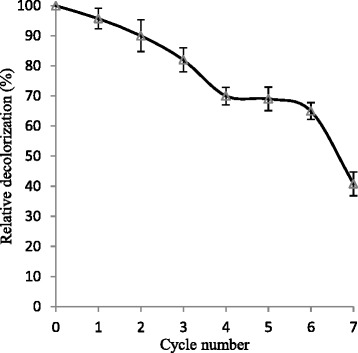
Reusability of free and Ca-alginate beads immobilized MnP. Ca-alginate beads were used for 7 decolorization cycles of 12 h each. After each cycle, the beads were removed and washed with 50 mM Na-malonate buffer and replaced with fresh dye solution. The activity of freshly prepared beads in the first run was defined as 100 %. The reusability study was performed in triplicate

It was investigated whether the Ca-alginate-bound MnP could be successfully recycled in repeated batch operations. The reusability of Ca-alginate beads immobilized MnP was investigated up to seven cycles and decolorization results are depicted in Fig. 8. The immobilized MnP retained more than 60 % of its initial decolorization activity after five repeated cycles and 40 % even after seven cycles. The gradual activity decline in the subsequent cycles could be correlated with enzyme inactivation. Upon frequent decolorization cycles, the substrate or product might cause blocking of some pores of beads that limit the access of dyes to the active site of entrapped MnP. However, Daassi et al. [4] highlighted that leakage of enzyme from alginate beads during washing after each cycle may lead to diminished activity. Similar observations of reusability have been documented previously [13]. The findings confirmed that Ca-alginate beads immobilization appears as excellent strategy for MnP immobilization with greater efficiency and reusability for removal of toxic dyestuffs.

#### Water quality parameters of MnP treated dyes

**Fig. 9:**
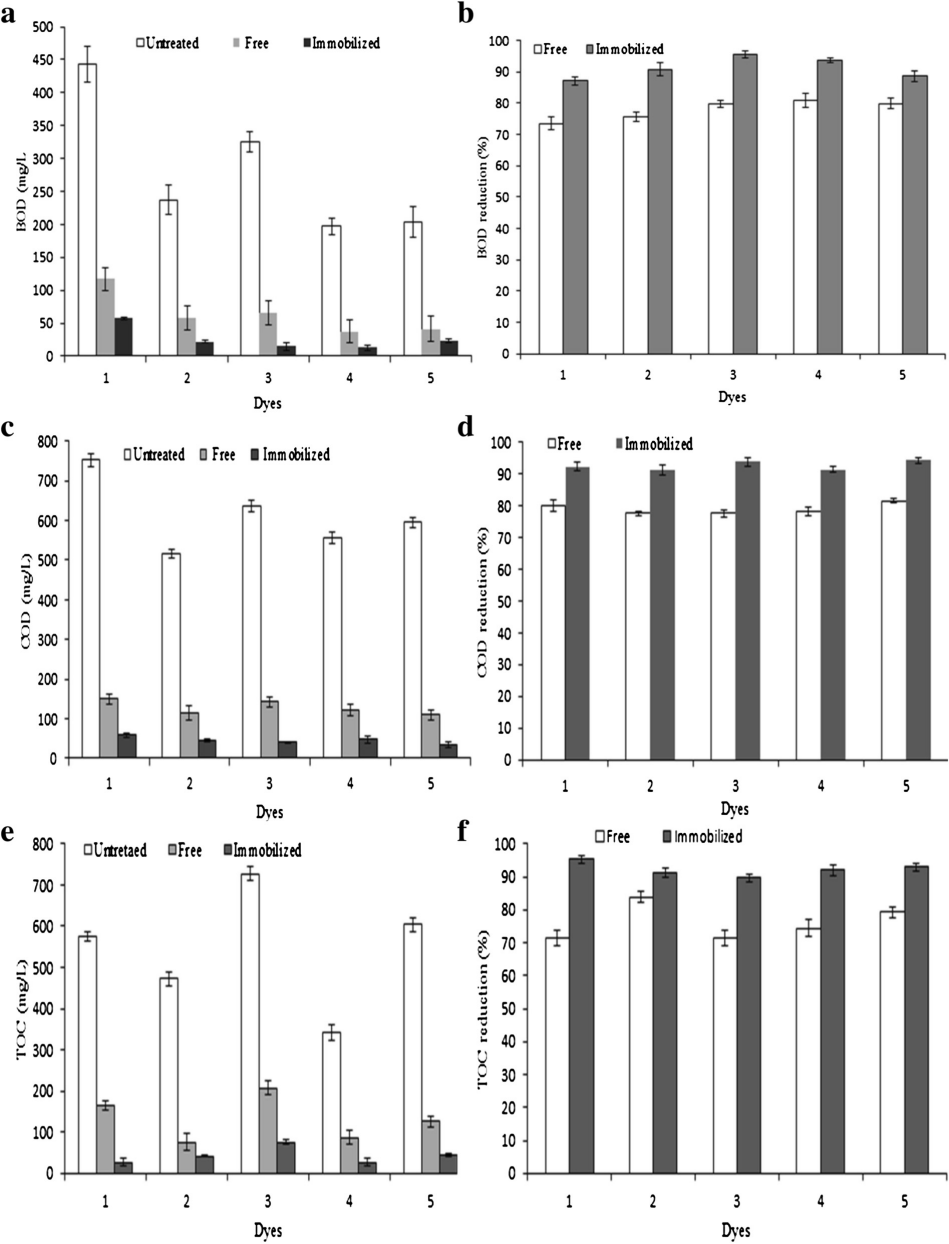
BOD, COD, TOC values of dyes (un-treated, free-MnP and immobilized MnP treated (mg/L)) and percentage reductions (*1*) Sandal-fix Red C_4_BLN (*2*) Sandal-fix Turq Blue GWF (*3*) Sandal-fix Foron Blue E_2_BLN (*4*) Sandal-fix Black CKF (*5*) Sandal-fix Golden Yellow CRL. BOD-biological oxygen demand, COD-chemical oxygen demand, TOC-total organic carbon

The dye degrading efficacy of both free and Ca-alginate immobilized MnP was assessed on the basis of BOD, COD and TOC reduction, as shown in Fig. 9. It was observed that, the characteristic values of untreated tested dyes solution were beyond the permissible range set by the National Environmental Quality Standards (NEQS) for the safe discharge into sewage treatment facilities [37]. Biochemical (or Biological) oxygen demand (BOD) is a measure of how rapidly biological organisms consume oxygen in a water body. It can be considered as an indication of the quality of a water source. Before treatment, the BOD values were found in the range of 197.28-443.21 mg/L for Sandal reactive dyes that reduced significantly after treatment with free and Ca-alginate immobilized MnP (Fig. 9a, b) BOD values of treated samples were in the range of permissible NEQ limits.

Chemical oxygen demand (COD) value is a useful measure of water quality that indicates the oxygen concentration required to oxidize all carbon compounds in a solution and is commonly used as an indirect measure of the amount of organic compounds present in wastewater. Figure 9c, d indicates that the COD values were considerably reduced in enzyme treated dye samples. High COD reduction in the treatment of textile effluent by *Pseudomonas* species has also been reported [38] while, Pourbabaee et al. [39] also observed similar reductions in COD during bio-treatment of textile effluent by a newly isolated *Bacillus* sp. Previously, Agarry and Ayobami [40] reported that *Pseudomonas fluorescence*, P*seudomonas nigificans* and *Pseudomonas gellucidium*, *Aspergillus niger*, *Proteus morganii* and *Fusarium compacticum* strains had good potential to remove color and degrade dyes, reduce COD and BOD levels between 39-48,74-97 and 77-95 %, respectively of the textile waste effluents with percent color removal.

A significant (p ≥ 0.05) decrease in TOC values of treated dyes demonstrated the effectiveness of biological treatment to transform large xenobiotic recalcitrant molecule of dyes into simpler fragments. The percentage reductions in TOC were found to be 71.31-83.88 % for free MnP, which increased to 89.58-95.29 % for Ca-alginate immobilized MnP.

#### Cytotoxicity reduction

**Fig. 10:**
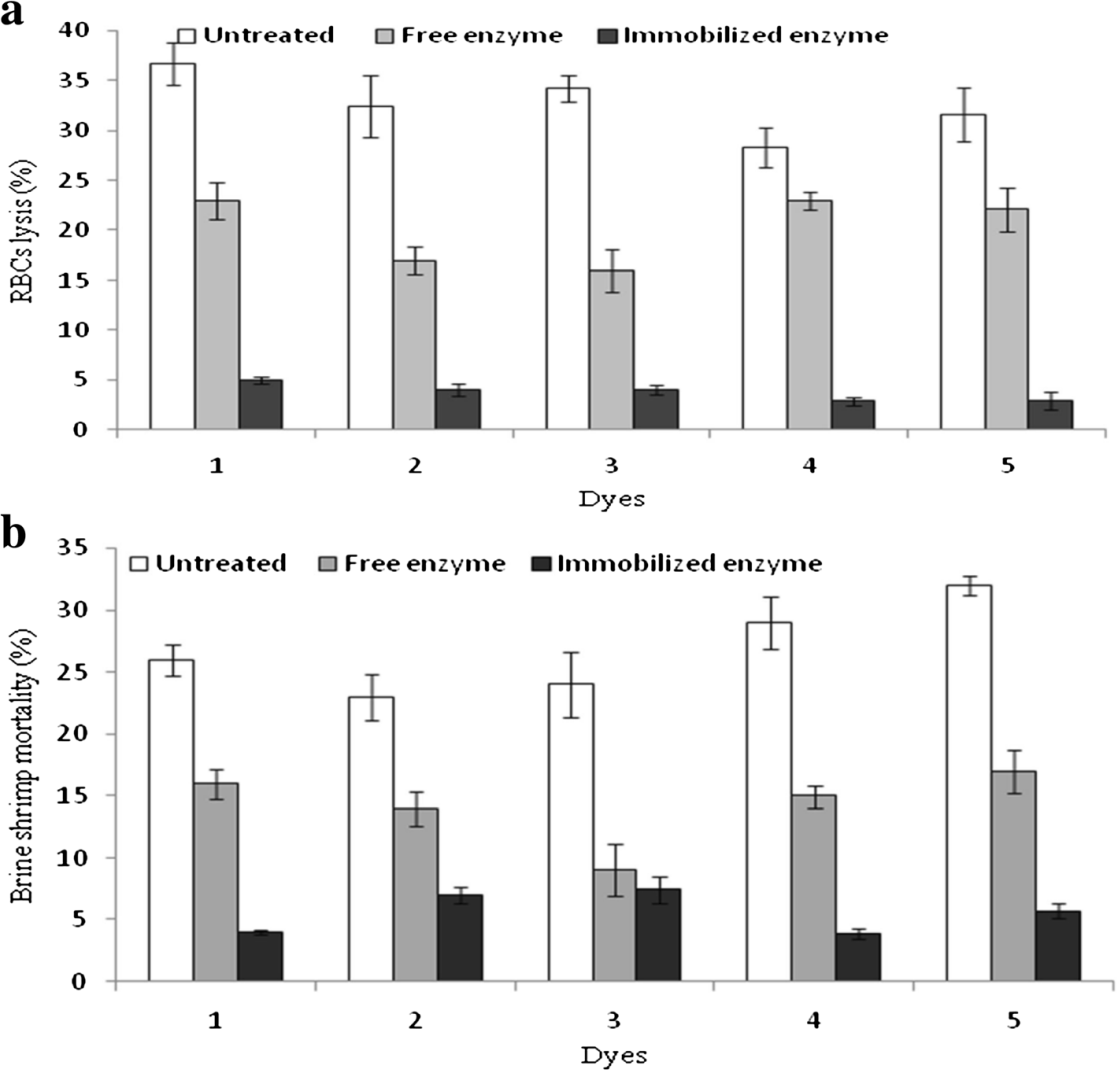
Cytotoxicity of reactive dyes treated with free and Ca-alginate beads immobilized MnP. For heamolytic test, positive and negative controls were Triton X-100 (0.1%) and phosphate buffer saline, respectively. For shrimp test, PC and NC were cyclophosphamide (10 g/mL) and sea water, respectively. The percentage increase or decrease of any parameters was calculated by computing the values before and after treatment

The cytotoxicity tests (heamolytic and brine shrimp lethality) were performed to evaluate the biological usefulness of free versus Ca-alginate immobilized MnP, since these tests are frequently used for the toxicity screening of pollutants (air, soil and water) [5]. Before treatment, the cytotoxicity of dyes was in the range of 28.3-36.7 % and 23-29 % for erythrocyte lysis and brine shrimp, respectively. As illustrated in Fig. 10, after treatment with free MnP, the cytotoxicity values were in the range of 16-23 % for erythrocytes lysis and 9-17 % for brine shrimp. In case of Ca-alginate immobilized MnP, the cytotoxicity was reduced to 2.9-5 % for erythrocytes lysis and 3.9-7.4 % for brine shrimp, respectively. Several methods have been employed for the decolorization of dyes successfully; however, the toxicity is still a consideration of active research area because residues/degradation intermediates and end product might be more toxic than the parent compound [41]. It was confirmed that the dyes were not only decolorized but also detoxified by the action of immobilized MnP. Previously; up to 98 % toxicity reduction of industrial effluents has been observed after treatment with WRF ligninolytic enzymes [42–44].

## Conclusions

Ca-alginate beads served as an excellent supporting matrix for *G. lucidum* MnP immobilization. The immobilized MnP exhibited highest activity at pH 4 and 60 °C. The improved thermal stability, reusability and high activity presented by Ca-alginate immobilized MnP would be the encouraging features. Besides, alginate exhibits many desirable characteristics, viz. biodegradability and biocompatibility, high gelling-ability, inexpensive and non-toxicity. Furthermore, subsequent exploitation of immobilized MnP for decolorization and detoxification of different textile reactive dyes makes it more valuable enzyme for various industrial applications. More efficient ligninolytic enzymes can be developed using advanced molecular or enzyme immobilization approaches to develop robust, stable and recyclable enzymes based technology for bioremediation of industrial effluents.

## Abbreviations

MnP: Manganese peroxidase
WRF: White rot fungi
PDA: Potato dextrose agar
H_2_O_2_: Hydrogen peroxide
BSA: Bovine serum Albumin
kDa: Kilo Dalton
SDS: Sodium dodecylsulphate
SSF: Solid state fermentation
DEAE-cellulsoe: Diethyl amino ethyl cellulose
TEMED: N, N, N΄, N΄-tetra-methylethylenediamine
(CaCl_2_. 2H_2_O): Calcium chloride dihydrate
BOD: Biochemical oxygen demand
COD: Chemical oxygen demand
TOC: Total organic carbon
NEQS: National Environmental Quality Standards
